# Predicting surgical resource consumption and in-hospital mortality in resource-scarce conflict settings: a retrospective study

**DOI:** 10.1186/s12873-021-00488-2

**Published:** 2021-08-11

**Authors:** Måns Muhrbeck, Zaher Osman, Johan von Schreeb, Andreas Wladis, Peter Andersson

**Affiliations:** 1 Department of Surgery in Norrköping, and Department of Biomedical and Clinical Sciences, Linköping University, Linköping, Sweden; 2 Center for Disaster Medicine and Traumatology, University Hospital, Linköping, Sweden; 3 International Committee of the Red Cross, Geneva, Switzerland; 4 Department of Global Public Health, Karolinska Institutet, Stockholm, Sweden; 5 International Medical Programme, Center for Disaster Medicine and Traumatology, University Hospital, Linköping, Sweden

**Keywords:** Armed conflicts, Health resources, Penetrating wounds

## Abstract

**Background:**

In armed conflicts, civilian health care struggles to cope. Being able to predict what resources are needed is therefore vital. The International Committee of the Red Cross (ICRC) implemented in the 1990s the Red Cross Wound Score (RCWS) for assessment of penetrating injuries. It is unknown to what extent RCWS or the established trauma scores Kampala trauma Score (KTS) and revised trauma score (RTS) can be used to predict surgical resource consumption and in-hospital mortality in resource-scarce conflict settings.

**Methods:**

A retrospective study of routinely collected data on weapon-injured adults admitted to ICRC’s hospitals in Peshawar, 2009–2012 and Goma, 2012–2014. High resource consumption was defined as ≥3 surgical procedures or ≥ 3 blood-transfusions or amputation. The relationship between RCWS, KTS, RTS and resource consumption, in-hospital mortality was evaluated with logistic regression and adjusted area under receiver operating characteristic curves (AUC). The impact of missing data was assessed with imputation. Model fit was compared with Akaike Information Criterion (AIC).

**Results:**

A total of 1564 patients were included, of these 834 patients had complete data. For high surgical resource consumption AUC was significantly higher for RCWS (0.76, 95% CI 0.74–0.78) than for KTS (0.53, 95% CI 0.50–0.56) and RTS (0.51, 95% CI 0.48–0.54) for all patients. Additionally, RCWS had lower AIC, indicating a better model fit. For in-hospital mortality AUC was significantly higher for RCWS (0.83, 95% CI 0.79–0.88) than for KTS (0.71, 95% CI 0.65–0.76) and RTS (0.70, 95% CI 0.63–0.76) for all patients, but not for patients with complete data.

**Conclusion:**

RCWS appears to predict surgical resource consumption better than KTS and RTS. RCWS may be a promising tool for planning and monitoring surgical care in resource-scarce conflict settings.

**Supplementary Information:**

The online version contains supplementary material available at 10.1186/s12873-021-00488-2.

## Background

In armed conflicts, civilian causalities often must rely on frail and fragmented health care systems that can only offer limited or no surgical resources to treat injured with potentially life-threatening injuries [1]. For healthcare providers delivering surgical care in conflicts, it is therefore imperative to know how to use available surgical resources as efficiently as possible. The extent of surgical resource consumption or workload in resource-scarce conflict settings is often discussed in terms of the number of surgical procedures, amputations, use of blood products, and mortality rate [2–7]. However, to our knowledge, no method has been validated to predict these outcomes in conflict settings. Trauma scores developed and used in non-conflict trauma settings to predict mortality could potentially be used for this purpose. The vital sign-based Revised Trauma Score (RTS) has been shown to predict need for surgery, haemorrhage, and mortality [8–11]. In resource-scarce trauma settings the Kampala Score (KTS), a simplified combination of age, RTS and the anatomical-based Injury Severity Score (ISS), has been validated to predict the need for admission and mortality [12]. However, the predictive abilities for RTS and KTS could be diminished in conflicts due to disintegrated infrastructure, lack of transportation means, non-existing prehospital care and long distance to hospital that cause a survival bias where injured with life-threatening, but treatable, injuries die before reaching the hospital. Furthermore, injury patterns in armed conflicts are different from those seen in civilian traumas. Penetrating injuries are more prevalent, whereas blunt injuries are more frequent in civilian traumas [13, 14].

The International Committee of the Red Cross (ICRC) is a humanitarian organization providing medical assistance to victims of war and other situations of violence, including independently running or supporting hospitals. In the early 1990s Mr. Robin Coupland, at the time Chief Surgeon for ICRC, developed a system for wound classification (Red Cross Wound Score, RCWS) in order to help surgeons systematically describe penetrating wounds in conflicts [15]. To classify a wound according to RCWS, the extent of tissue damage (RCWS grade) and type of tissue involved (RCWS type) must be assessed (Table 1). The RCWS grade corresponds to amount of energy transferred to the tissue at time of injury [4, 15]. Studies have demonstrated that RCWS grade is associated with need of surgery, number of surgical procedures, in-hospital amputation, and mortality [4, 16–19]. However, RCWS predictive ability regarding the extent of surgical resource consumption and mortality has not previously been examined.

**Table 1:** Parameters included in the Red Cross Wound Score (RCWS) [4]

Wound feature	Definition
E (entry)	Entry wound in cm
X (exit)	Exit wound in cm (X = 0 if no exit)
C (cavity)	Can the cavity of the wound take two fingers before surgical excision?
	C0 = No
	C1 = Yes
F (fracture)	Fracture
	F0 = No fracture
	F1 = Simple fracture, hole or insignificant comminution
	F2 = Clinically significant comminution
V (vital structure)	Injury threatening life or threatening life or limb
	V0 = No vital structure injured
	VN = (neurological) penetration of the dura of the brain or spinal cord
	VT = (thorax or trachea) penetration of the pleura or of the larynx/trachea in the neck
	VA = (abdomen) penetration of the peritoneum
	VH = (haemorrhage) injury of a major peripheral blood vessel, down to the brachial artery in the arm or the popliteal artery in the leg or carotid artery in the neck

The aim of the present study was therefore to determine if RCWS, KTS and RTS have predictive abilities to assess the extent of surgical resource consumption as well as in-hospital mortality in resource-scarce conflict settings. Surgical resource consumption was characterized by the number of surgical procedures, blood transfusions and limb amputation. A secondary aim was to assess if the individual components of RCWS are independent predictors of surgical resource consumption and in-hospital mortality. If any of these scores could be found to be reliable, they would provide an urgently needed instrument for planning and monitoring surgical interventions in resource-scarce conflict settings.

## Patients and settings

Routinely collected patient data from ICRC’s hospitals in Peshawar, Pakistan, and Goma, the Democratic Republic of the Congo (DRC), were retrospectively analysed for this study. Patients treated at ICRC’s hospital in Peshawar were predominantly injured in the conflict on both sides of the Pakistani–Afghanistan border. The hospital was closed in 2014 and had 116 beds, a critical care unit, three operating theatres, X-ray services and a laboratory [20]. Patients treated at the hospital in Goma were mainly injured in the ongoing conflict between several rivalling factions in the Kivu province in the DRC. Compared to the conflict along the Afghanistan–Pakistani border, less injures from indiscriminate weapons such as grenades, bombs and anti-personnel mines have been reported [2, 20, 21]. The hospital is still operational and has 65 beds, one operating theatre, X-ray services and a laboratory. Both hospitals had access to physiotherapy for post-operative mobilization and rehabilitation with prosthesis fitting [20].

At both hospitals combatants as well as local citizens with weapon-related injuries were treated. Those seeking care were not asked whether they were civilians or combatants. Care at both hospitals was provided free of charge during the study period.

## Method

The material consists of two matching databases of anonymized patient data from the ICRC’s hospitals in Peshawar and Goma. All patients treated between 18 February 2009–9 May 2012 (Peshawar) and 17 November 2012–17 September 2014 (Goma) were continuously included in the two databases (Excel® spreadsheet) at the time of patient discharge. Entries in the Peshawar database were done by two physicians whose primary job was to maintain the database. Entries in the Goma database were done by health care staff after receiving basic training. In total 3028 patients were recorded in the Peshawar database and 689 in Goma database. The two databases were merged and transferred to SPSS Statistical software version 25 (IBM Corporation, Armonk, NY, USA) for this study. The final database was validated with respect to the original databases by comparing data from 50 patients from each database without any differences identified. Patients that fulfilled the following inclusion criteria were extracted from the merged database: adult patients with weapon-related penetrating injuries, assessed RCWS and known discharge status. Adults were defined as age 15 or older in accordance with previous studies from similar settings [20, 22]. RCWS was determined by the surgeon at the time of the first surgical procedure according to ICRC guidelines [4]. Surgeons had received training in the use of RCWS prior to deployment. The wound with the highest RCWS grade and type was recorded as the first injury. To categorize a penetrating injury according to RCWS, the wound’s grade 1–3 (wound’s entry- and exit-diameter and if a two-fingers- wide cavity exists) and type (soft tissue, bones, threatening life or threatening life or limb) needs to be determined (Table 1).

**Fig. 1:**
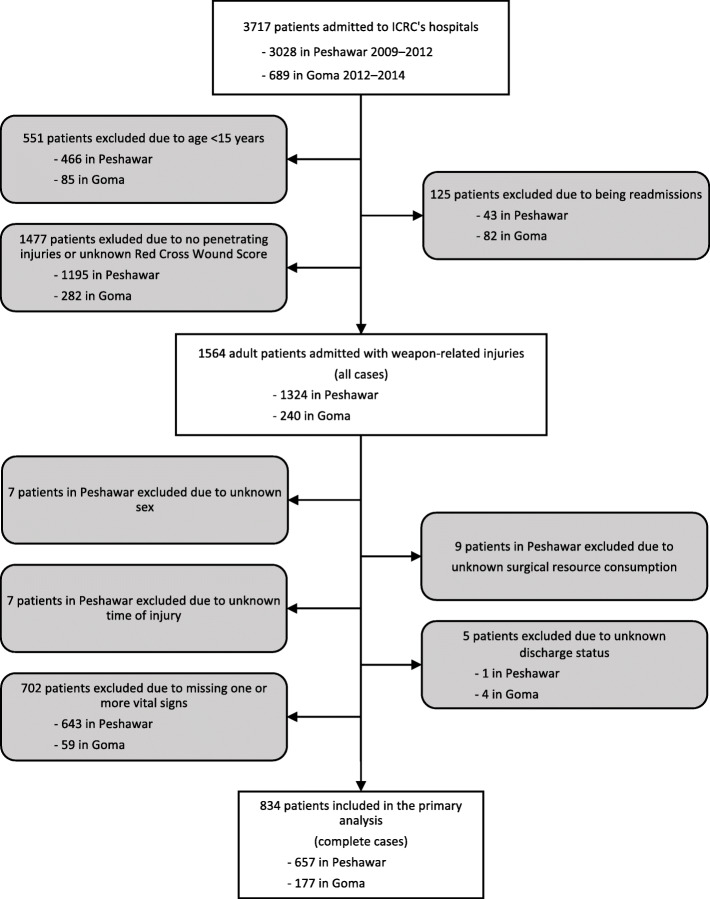
Flow chart of inclusion. All patients ≥15 years with weapon-related injuries classified with Red Cross Wound Score (RCWS), known surgical resource consumption and discharge status were included

Depending on the grade and type, a wound is assigned to one of twelve different categories (Additional Table 1) [4, 15, 23]. The presence of a second severe injury was dichotomously registered as existing or not. Severe injury was defined as a cavitating soft tissue injury with entry or exit 10 cm or more, or open fracture or injury to vital structure. This definition excludes RCWS soft tissue injury grade 1 and 2 and translates to an Abbreviated Injury Score of 2 or above for any given anatomical site, corresponding to the definition used in KTS [24]. In the case of multiple admissions only the first one was included. Patients with unspecified sex, time of injury and vital signs were considered to be incomplete cases and excluded in the primary analysis (Fig. 1).

ICRC characterizes surgical workload in conflict settings by severity of injuries, number of operations per patient, number of blood transfusions required and length of hospitalization [4]. To better reflect the actual surgical resource consumption, in the present study, this definition was modified to: number of surgical procedures under anaesthesia, blood transfusions and limb amputation. Length of hospitalization was excluded as this can be influenced by issues that are not related to the treatment of the patients’ injuries. This modification was done after advice from senior ICRC surgeons, and is consistent with how surgical resource consumption is characterized in previous studies [2–5, 7, 25].

High surgical resource consumption was dichotomously defined as:

1. Three or more surgical procedures under anaesthesia OR
2. Three or more blood transfusions OR
3. In-hospital amputation proximal to the metacarpophalangeal joints of the hand or the midtarsal joints of the foot.

Three or more surgical procedures were chosen because a study with 16,172 patients treated at ICRC’s hospitals demonstrated that 66% of the patients were treated with two surgical procedures or fewer [4]. Three or more blood transfusions were chosen as a previous ICRC-study with 4470 weapon-injured patients demonstrated that 15.5% were transfused and an average of 2.9 units was given [25]. Furthermore, the ICRC recommends an availability of 100 units/100 patients if anti-personnel mines are used in the conflict [4]. In-hospital limb amputation was included as these patients require extensive resources postoperatively in terms of physiotherapy and prosthesis fitting. In-hospital mortality was defined as deceased during hospital stay.

### Statistical methods

We estimated that 592 patients would be needed to detect a difference of 0.10 in the trauma scores’ ability to detect high surgical resource consumption. A reference area under the receiver operating characteristic curve (AUC) of 0.75, confidence interval (CI) 95% and power 90% was used for this sample size calculation. Nonetheless, all eligible patients were included in the analysis.

Descriptive comparisons between the hospitals in Peshawar and Goma were analysed using Fisher’s exact test for categorical variables and Mann-Whitney *U*-test for continuous variables. Values are given as mean with standard deviation (SD) where appropriate. To compare the predictive effect of the individual components of RCWS, KTS and RTS on surgical resource consumption and in-hospital mortality, binary logistic regression was performed. Sex, age (15–49 and > 49 yrs.), time since injury (0–6, 7–24 and > 24 h) systolic blood pressure (0–75, 76–89, and > 89 mmHg), respiratory rate (≤9, 10–29, ≥30 /min), Glasgow Coma Scale (≤5, 6–8, 9–12 and 13–15), RCWS grade, RCWS type and the existence of more than one severe injury were predictors included in the regression analysis as these are the components of RCWS, KTS and RTS or have in previous studies been identified as influencing the need for surgical intervention, blood transfusion or amputation [2, 26–29]. Vital signs were taken upon admission, and the intervals used match the intervals used in RTS [8]. The procedure to calculate RTS and KTS has been described elsewhere [8, 12]. Forward Wald was used to calculate a final regression model. When comparing differences in resource consumption for the Peshawar and Goma hospital, hospital site was added to the final regression model. The impact of predictors is presented as odds ratio (OR) with 95% confidence intervals. *P*-values (two-tailed) less than 0.05 were considered significant for both the univariate and logistic regression analysis.

The sensitivity and specificity associated with the ability of RCWS, KTS and RTS to predict high surgical resource consumption and in-hospital mortality was assessed by analysing of the AUCs. To enable analysis of patients with more than one severe injury, RCWS was adjusted for the existence of additional severe injuries. In making comparisons of the prognostic abilities of RCWS, KTS and RTS regarding high surgical resource consumption and in-hospital mortality the AUCs along with 95% CI were used. Overlapping confidence intervals were interpreted as indicating non-significant differences in the prognostic abilities of RCWS, KTS and RTS.

Comparison of goodness of model fit for RCWS, KTS and RTS in predicting high surgical resource consumption and in-hospital mortality was analysed by Akaike Information Criterion (AIC). A lower AIC score for a trauma score indicates a better fitting model in comparison with the other evaluated trauma scores. Little’s MCAR test and imputation with fully conditional specification was done to enable the analysis of incomplete cases and thereby assessing the impact of exclusion of patients with unknown sex, time of injury and vital signs. Mann-Whitney *U*-test with Fisher’s exact test was used to evaluate why a patient ended up in the high resource group.

## Results

### Descriptive

A total of 657 of 1324 patients treated in Peshawar and 177 of 240 patients treated in Goma had complete data (Complete cases, Fig. 1). There were more men and older patients injured in Peshawar compared to Goma, and more patients had injuries from indiscriminate weapons such as bombs, shells and fragments or anti-personnel mines, and fewer from gunshots (*p* ≤ 0.001 for all) (Table 2).

**Table 2:** Mechanism of injury for patients treated at ICRC’s hospitals in Peshawar and Goma

Mechanism	Peshawar Total = 657 n (% of total)	Goma Total = 177 n (% of total)	*P* value
Gunshot	357 (54.3)	156 (88.1)	< 0.001
Bomb, shell, fragment	266 (40.5)	17 (9.6)	< 0.001
Anti-personnel mine	31 (4.7)	0	0.001
Arme blanche	1 (0.2)	3 (1.7)	0.031
Other	2 (0.3)	0	
Unknown	0	1 (0.6)	

There was no difference in the RCWS type for the first injury recorded between the hospitals. However, patients in Goma were more likely to be admitted within 6 hours of their injury or have injuries to the upper limbs, while patients in Peshawar more frequently had injuries to the head, face or more than one severe injury (*p* < 0.05 for all) (Table 3).

**Table 3:** Injury patterns for patients treated at ICRC’s hospitals in Peshawar and Goma

Injury patterns	Peshawar Total = 657 n (% of total)	Goma Total = 177 n (% of total)	*P* value
Location of injury^a^			
Head or face	58 (8.8)	6 (3.4)	0.016
Neck	15 (2.3)	1 (0.6)	0.216
Thorax	69 (10.5)	13 (7.3)	0.255
Back	39 (5.9)	5 (2.8)	0.128
Abdomen	30 (4.6)	13 (7.3)	0.178
Pelvis^b^	26 (4.0)	9 (5.1)	0.527
Upper limbs	136 (20.7)	50 (28.2)	0.042
Lower limbs	276 (42.0)	76 (43.0)	0.864
Unknown	8 (1.2)	4 (2.3)	0.294
RCWS type^a^			
Soft tissue	225 (34.2)	62 (35.0)	0.859
Fracture	269 (40.9)	75 (42.4)	0.732
Penetration of peritoneum	38 (5.8)	17 (9.6)	0.086
Penetration of pleura^c^	47 (7.2)	9 (5.1)	0.399
Penetration of dura^d^	34 (5.2)	5 (2.8)	0.231
Major periph. blood vessel^e^	44 (6.7)	9 (5.1)	0.492
Additional severe injuries^f^			0.005
No	562 (85.6)	165 (93.2)	
Yes	95 (14.4)	12 (6.8)	

^a^First injury recorded; ^b^Pelvis including buttocks, perineum, and genitals; ^c^Including the larynx/trachea of the neck; ^d^The dura of the brain or spinal cord; ^e^ Down to the brachial or popliteal or carotid arteries; ^f^Corresponding to Abbreviated Injury Score ≥ 2

Chest-tube insertion and fracture surgery were more frequent in Peshawar than in Goma (*p* = 0.039 and *p* < 0.001, respectively). There was no difference in frequencies of amputations, surgical procedures, blood transfusions and in-hospital mortality between the hospitals (Table 4).

**Table 4:** Surgical care and in-hospital mortality for patients treated at ICRC’s hospitals in Peshawar and Goma

Surgical care and in-hospital morality	Peshawar Total = 657		Goma Total = 177		
	n (% of total)	Mean (SD)	n (% of total)	Mean (SD)	*P* value
Surgical procedure under anesthesia^a^					
Craniotomy	21 (3.2)		2 (1.1)		0.195
Thoracotomy	8 (1.2)		0		0.214
Chest-tube	67 (10.2)		9 (5.1)		0.039
Laparotomy	82 (12.5)		14 (7.9)		0.111
Peri. vasc. Repair	31 (4.7)		4 (2.3)		0.148
Arm-amputation	11 (1.7)		3 (1.7)		1
Leg-amputation	34 (5.2)		5 (2.8)		0.122
Any amputation	38 (5.8)	0.06 (0.23)	8 (4.5)	0.05 (0.21)	0.513^d^
Any frac. surgery^b^	157 (23.9)	0.24 (0.43)	18 (10.2)	0.10 (0.30)	< 0.001^d^
Any soft tissue surgery^c^	608 (92.5)	0.93 (0.26)	170 (96.0)	0.96 (0.20)	0.098^d^
Any type of procedure	637 (97.0)	0.97 (0.17)	168 (94.9)	0.95 (0.22)	0.188^d^
Blood transfusion		0.19 (0.56)		0.34 (1.19)	0.521^d^
0 units	543 (82.6)		153 (86.4)		0.356
1–2	75 (11.4)		15 (8.5)		0.278
≥ 3	34 (5.2)		9 (5.1)		1
Unknown	5 (0.8)		0		0.590
In-hospital mortality		0.03 (0.16)		0.02 (0.13)	0.431^d^
No	639 (97.3)		174 (98.3)		
Yes	18 (2.7)		3 (1.7)		

^a^Some patients have been subjected to several procedures which results in more procedures than patients and sum of percentages more than 100; ^b^External fixation, Kirchner wire, traction, or manipulation; ^c^Debridement, split skin graft, delayed primary closure, burn care, or change of dressing; ^d^*P*-value for comparison of mean

### Surgical resource consumption

The relationship between surgical resource consumption, sex, age, vital signs and RCWS are shown in Table 5. A larger portion of the patients with high surgical resource consumption had lower systolic blood pressure, higher RCWS grade, higher RCWS type and more frequently had more than one severe injury compared to patients with low surgical resource consumption (*p* < 0.01 for all). When adjusting for all predictors in the final logistic regression model RCWS grade, RCWS type and existence of more than one severe injury remained significant. Sex, age, time since injury, systolic blood pressure, respiratory rate and Glasgow Coma Scale did not affect a patient’s surgical resource consumption.

**Table 5:** Relationship between surgical resource consumption, sex, age, vital signs and Red Cross Wound Score (RCWS)

Surgical resource consumption	Univariate analysis Complete cases Total = 834			Logistic regression analysis Complete cases Total = 834			
	Low Total = 472 n (%)	High^a^ Total = 362 n (%)	*P* value	All confounders 1 = High^a^ Odds ratio (95% CI)	*P* value	Final model (Wald) 1 = High^a^ Odds ratio (95% CI)	*P* value
Sex			1.000				
Male	418 (88.6)	320 (88.4)		1			
Female	54 (11.4)	42 (11.6)		1.14 (0.68–1.90)	0.626		
Age			0.710				
15–49 years	433 (91.7)	329 (90.9)		1			
> 49	39 (8.3)	33 (9.1)		1.23 (0.70–2.17)	0.466		
Time since injury			0.152				
0–6 h	15 (3.2)	21 (5.8)		1			
7–24 h	203 (43.0)	159 (43.9)		0.68 (0.30–1.54)	0.352		
> 24 h	254 (53.8)	182 (50.3)		0.66 (0.29–1.52)	0.327		
Systolic blood pressure			0.004				
> 89 mmHg	464 (98.3)	341 (94.2)		1			
76–89	5 (1.1)	10 (2.8)		1.16 (0.33–4.11)	0.931		
0–75	3 (0.6)	11 (3.0)		1.65 (0.44–6.20)	0.821		
Respiratory rate			0.446				
10–29/min	458 (97.0)	347 (95.9)		1			
≤ 9	–	–		–	–		
≥ 30	14 (3.0)	15 (4.1)		0.88 (0.37–2.09)	0.772		
Glasgow Coma Scale			0.077				
13–15	465 (98.5)	347 (95.8)		1			
9–12	4 (0.9)	10 (2.8)		1.09 (0.31–3.87)	0.896		
6–8	2 (0.4)	4 (1.1)		1.60 (0.22–11.65)	0.644		
≤ 5	1 (0.2)	1 (0.3)		0.84 (0.05–15.76)	0.906		
RCWS grade			< 0.001				
1 (simple)	266 (56.4)	83 (22.9)		1		1	
2 (medium)	185 (39.2)	178 (49.2)		2.60 (1.81–3.74)	< 0.001	2.63 (1.84–3.76)	< 0.001
3 (large)	21 (4.4)	101 (27.9)		11.60 (6.61–20.36)	< 0.001	11.94 (6.85–20.80)	< 0.001
RCWS type			< 0.001				
Soft tissue	220 (46.6)	67 (18.5)		1		1	
Fracture	164 (34.8)	180 (49.7)		2.39 (1.62–3.54)	< 0.001	2.32 (1.58–3.41)	< 0.001
Threatening life	59 (12.5)	45 (12.4)		2.16 (1.27–3.67)	0.005	2.20 (1.31–3.72)	0.003
Threatening life/limb	29 (6.1)	70 (19.4)		4.67 (2.61–834)	< 0.001	4.80 (2.77–8.33)	< 0.001
Additional severe injuries^b^			< 0.001				
No	439 (93.0)	288 (79.6)		1		1	
Yes	33 (7.0)	74 (20.4)		2.90 (1.83–4.89)	< 0.001	3.00 (1.85–4.88)	< 0.001

^a^Defined as ≥3 surgical procedures under anesthesia or ≥ 3 blood transfusions or limb amputation; ^b^Corresponding to Abbreviated Injury Score ≥ 2

**Fig. 2:**
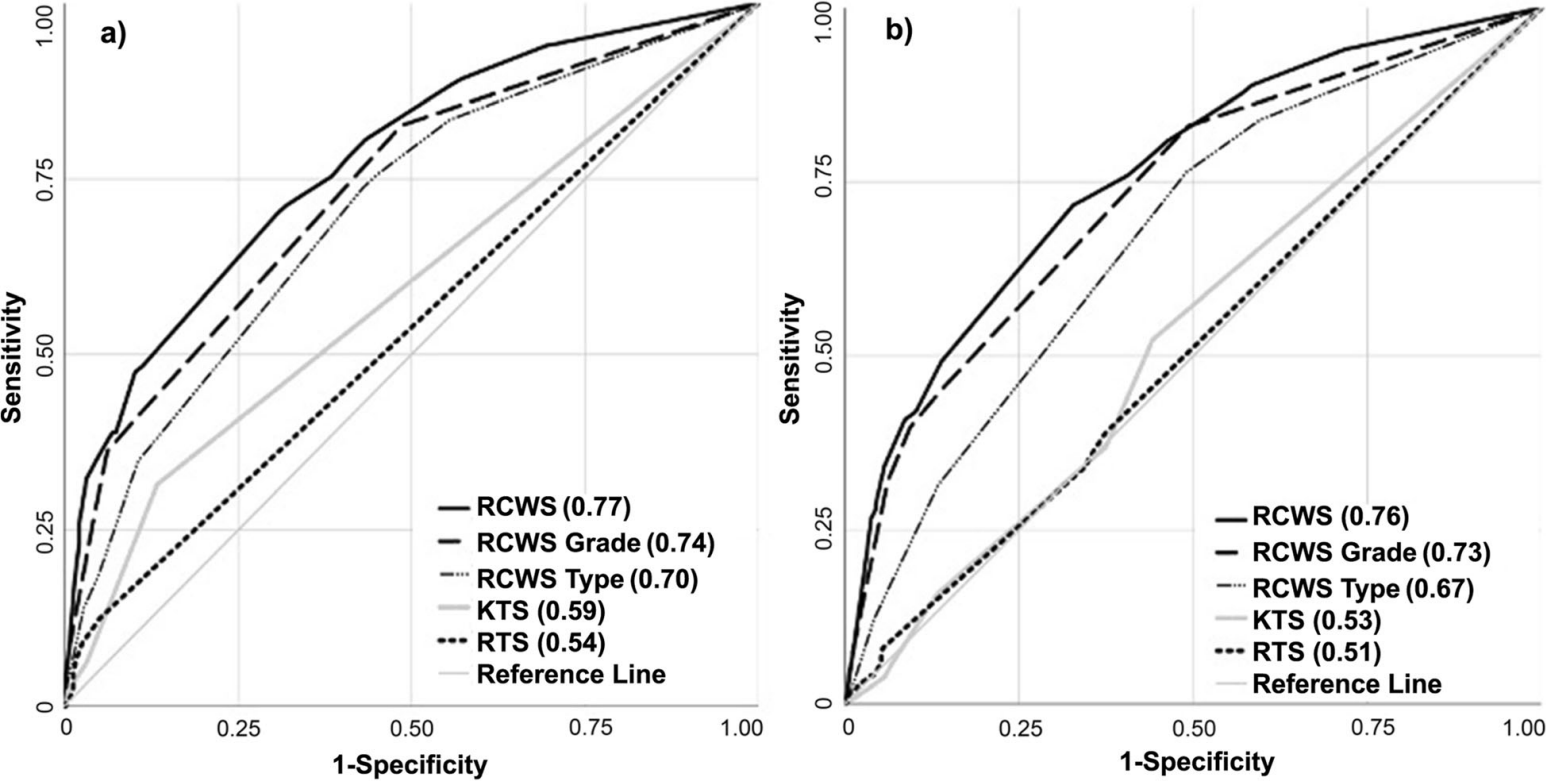
High surgical resource consumption by RCWS, KTS and RTS. AUC in parenthesis. Note that RCWS, RCWS grade and RCWS type were adjusted for existence of additional severe injuries, corresponding to Abbreviated Injury Score ≥ 2. **a** Complete cases (*n* = 834). **b** All cases using imputed data for incomplete cases (*n* = 1555. 9 patients excluded due to unknown surgical resource consumption)

For complete cases RCWS achieved significantly higher AUC and lower AIC, i.e., better predictive ability and model fit, than KTS and RTS for high surgical resource consumption (Table 6, Fig. 2). In the logistic regression model for all cases, using imputed data for incomplete cases, the same predictors plus age more than 49 years (OR 1.84, 95% CI 1.26–2.70, *p* < 0.001) were found to be significant (Additional Table 2). There was no statistically significant difference in the AUC for all cases compared to complete cases (Table 6 and Fig. 2).

**Table 6:** Analysis of AUC and AIC for individual scores for high surgical resource consumption

High surgical resource consumption	Complete cases Total = 834		All cases^a^ Total = 1555^b^	
	AUC (95% CI)	AIC	AUC (95% CI)	AIC
RCWS^c^	0.77 (0.74–0.81)	904	0.76 (0.74–0.78)	1777
- Grade^c^	0.74 (0.70–0.77)	948	0.73 (0.71–0.76)	1833
- Type^c^	0.70 (0.67–0.74)	1010	0.67 (0.65–0.70)	1979
KTS	0.59 (0.55–0.63)	1114	0.53 (0.50–0.56)	2072
RTS	0.54 (0.50–0.58)	1145	0.51 (0.48–0.54)	2096

^a^Using imputed data for incomplete cases; ^b^9 patients excluded due to unknown surgical resource consumption; ^c^Adjusted for existence of additional severe injuries, corresponding to Abbreviated Injury Score ≥ 2

### Subgroup analysis of high resource consumption and hospital site

When hospital site was added to the final logistic regression model of complete cases, patients treated in Goma were more likely to have a high surgical resource consumption than patients treated in Peshawar (OR 2.01, 95% CI 1.36–2.98, *p* < 0.001). After adjusting surgical procedures shown in Table 3 for the total number of injured sites, patients in Goma were more frequently exposed to soft tissue surgery (Goma: mean 3.58 times/patient, SD 3.39, and Peshawar: 2.18, SD 1.75, *p* < 0.001) and also to any type of surgical procedures under anaesthesia than in Peshawar (Goma: mean 3.71 times/patient, SD 3.71, and Peshawar: 1.96, SD 1.55, *p* < 0.001). When comparing Goma and Peshawar regarding patients with high surgical resource consumption the distribution between hospitals was similar regarding the number of surgical procedures, blood transfusions and amputation rates. Three or more surgical procedures most strongly explained why most patients ended up in the high resource consumption group for both hospital sites (Goma: 86 of 102 patients, 84.3%, and Peshawar: 197 of 260 patients, 75.8%, *p* = 0.09).

### In-hospital mortality

**Fig. 3:**
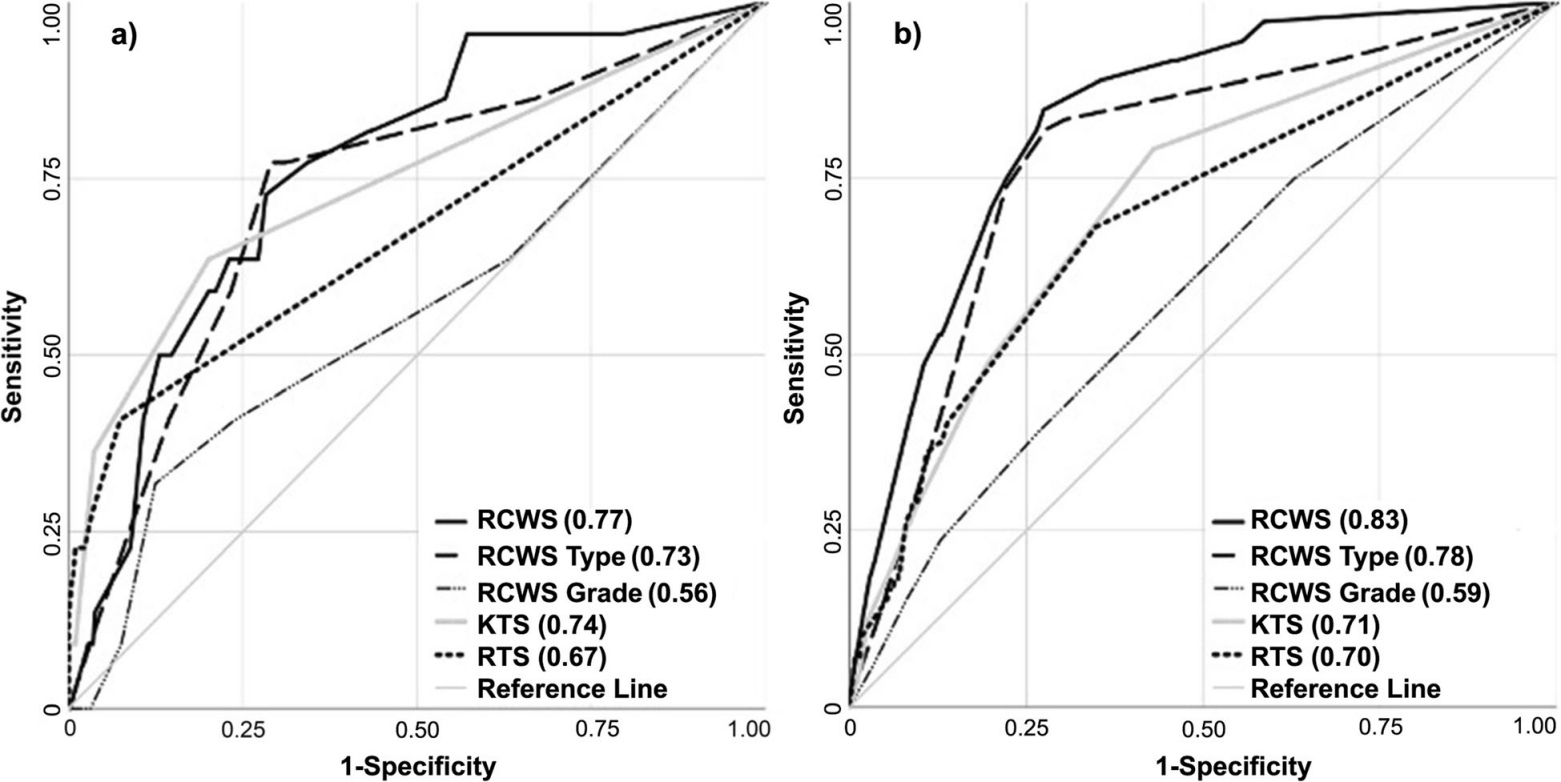
In-hospital mortality by RCWS, KTS and RTS. AUC in parenthesis. Note that RCWS, RCWS grade and RCWS type were adjusted for existence of additional severe injuries, corresponding to Abbreviated Injury Score ≥ 2. **a** Complete cases (*n* = 834). **b** All cases using imputed data for incomplete cases (*n* = 1559. 5 patients excluded due to unknown discharge status)

For in-hospital mortality, there was no difference between the hospitals (Peshawar: 18 patients, 2.7% and Goma: 3 patients, 1.7%, *p* = 0.781). At both hospitals, a larger portion of the patients who died in hospital arrived sooner after injury, had systolic blood pressure < 90 mmHg, GCS < 13 and higher RCWS type than those discharged alive (*p* < 0.05 for all) (Additional Table 3). In the logistical regression model for complete cases, no predictors were significant for in-hospital mortality. RCWS AUC and AIC for in-hospital mortality was not significantly better than KTS and RTS (Table 7, Fig. 3).

**Table 7:** Analysis of AUC and AIC for individual scores for in-hospital mortality

In-hospital mortality	Complete cases Total = 834		All cases^a^ Total = 1559^b^	
	AUC (95% CI)	AIC	AUC (95% CI)	AIC
RCWS^c^	0.77 (0.67–0.86)	180	0.83 (0.79–0.88)	481
- Grade^c^	0.56 (0.42–0.70)	176	0.59 (0.52–0.66)	582
- Type^c^	0.73 (0.62–0.84)	193	0.78 (0.73–0.84)	506
KTS	0.74 (0.62–0.87)	183	0.71 (0.65–0.76)	537
RTS	0.67 (0.54–0.81)	182	0.70 (0.63–0.76)	534

^a^Using imputed data for incomplete cases; ^b^5 patients excluded due to unknown discharge status; ^c^Adjusted for existence of additional severe injuries, corresponding to Abbreviated Injury Score ≥ 2

In the logistic regression model for all cases, using imputed data for incomplete cases, systolic blood pressure 76–89 mmHg and RCWS types threatening life and threatening life or limb were found to be significant predictors for in-hospital mortality (systolic blood pressure 76–89 mmHg: OR 4.63, 95% CI 1.59–13.44, threatening life: OR 10.72, 95% 4.42–26.01 and threatening life or limb: 8.43, 95% CI 3.36–21.18, *p* < 0.01 for all) (Additional Table 3). In the analysis of AUC for all cases RCWS demonstrated a significantly higher AUC and lower AIC, i.e. better predictive ability and model fit, than KTS and RTS for in-hospital mortality (Table 7 and Fig. 3).

## Discussion

To our knowledge, this is the first study to reveal that RCWS could have predictive abilities for high surgical resource consumption in adult patients with weapon-related injuries. This finding is consistent with previous studies that have demonstrated a relationship between RCWS and the need for surgery, number of procedures, in-hospital amputation and mortality [16–19]. Furthermore, in a recent study from our group findings suggest that RCWS grade is correlated with number of surgeries, blood transfusions and hospital stay in paediatric patients with weapon-related extremity wounds [30].

When comparing RCWS with KTS and RTS, commonly used trauma scores in non-conflict settings, RCWS had a better ability to predict high surgical resource consumption in conflict settings [11, 24, 31]. KTS and RTS were chosen for comparison as they have been demonstrated to be able to predict resource-related outcomes in trauma settings [10–12, 32, 33]. Furthermore, RCWS, KTS and RTS are similar in the aspect that they can be assessed without the need of advanced diagnostic equipment or calculations. A source of error is that we had a large number of missing vital signs in our dataset potentially affecting the predictive abilities for KTS and RTS. To address this issue, we performed the logistic regression model with imputed data for missing vital signs without any substantial changes in vital signs impact on surgical resource consumption.

Patients treated in Peshawar were more frequently injured by indiscriminate weapons, arrived later to the hospital and more often had more than one severe injury than in Goma. Therefore, it was unexpected to find that the patients in Goma more often required high surgical resources than in Peshawar. The reason for the observed difference in surgical resource consumption is unclear as both hospitals followed the same treatment protocol and were similarly equipped [20]. A possible explanation could be that patients treated in Goma were in a poorer general condition with impaired healing ability due to being more frequently affected by malnutrition or endemic diseases such as tuberculosis and malaria compared to Peshawar [34]. This difference in consumption of surgical resources between Goma and Peshawar illustrates how RCWS potentially could be used to systematically evaluate treatment facilities use of surgical resources in relation to injuries treated.

Consistent with other studies from armed conflicts, a low in-hospital mortality was observed at both hospitals [4, 20, 35]. In the analysis of all cases systolic blood pressure 76–89 mmHg, RCWS types threatening life and threatening life or limb were found to be significant predictors for in-hospital mortality rendering significantly better predictive ability for RCWS than KTS and RTS. The grade component of RCWS has previously been found to be associated with mortality in patients with conflict-related abdominal wounds with penetration of the peritoneum [19]. Otherwise, little has previously been known regarding RCWS predictive ability for mortality. In non-conflict settings better predictive abilities for KTS and RTS regarding mortality following weapon-related injuries have been reported [36, 37]. The worse predictive ability observed in our study could be explained by the survival bias where patients with the most altered vital signs die before reaching the hospital. The low in-hospital mortality and that over 90% of the patients in our material arrived more than 6 hours after the injury occurred supports this assumption. The conclusion that RCWS has better predictive ability for in-hospital mortality than KTS and RTS in conflicts must therefore be made with caution.

The difficulty to foresee what resources will be needed in conflicts coupled with fragile and unreliable supply chains can potentially lead to disastrous effects in terms of morbidity and mortality [4, 38]. Our findings support the use of RCWS as an instrument for evaluation of surgical resource needs and in-hospital mortality in relation to injuries treated. RCWS could consequently be used to ensure that the surgical resources provided meet the actual treatment needs. The systematic use of RCWS could also enable quality audits and research in conflict settings. The inclusion of RCWS in the minimum data set recommended by the Consensus Framework for the Humanitarian Surgical Response to 21st Century Warfare should therefore be considered [39]. However, prospective studies from different armed conflicts are needed to assess the external validity of our findings and determine the predictive ability for each RCWS category. Furthermore, the interobserver reliability for RCWS needs to be evaluated.

### Limitations

The use of routinely collected patient data limits the possibility of controlling for potentially confounding factors, such as variations in workload, adherence to treatment protocols, access to drugs, blood products, disposable material, and missing data. To mitigate the issue with missing data, we also analysed the predictive abilities for RCWS, KTS and RTS using an imputed dataset, with only minor alterations in the results. Nevertheless, alternative explanations for our findings could exist. A prospective cohort study design would have allowed for better control of confounding factors. However, prospective data collection in conflict settings is often difficult due to restraints in infrastructure and frequent rotation of health care staff. Additionally, participation in a study can have negative implications for participating health care staff and patients.

## Conclusion

Our findings indicate that RCWS was able to predict high surgical resource consumption better than and in-hospital mortality at least equal to KTS and RTS. In addition to facilitating systematic evaluation of penetrating injuries RCWS could consequently be a useful instrument in planning and monitoring of surgical care facilities in resource-scarce conflict settings. However, future studies are needed to determine RCWS predictive ability and interobserver variability in different conflicts and for different care providers.

## Supplementary Information

**Additional file 1: Table 1.** Explanation on how to classify penetrating injuries according to Red Cross Wound Score (RCWS).

**Additional file 2: Table 2.** Relationship between surgical resource consumption, sex, age, vital signs and Red Cross Wound Score (RCWS). Table of univariate and logistic regression analysis examining the relationship between surgical resource consumption, sex, age, vital signs, RCWS grade and type forpatients treated at ICRC’s hospitals in Peshawar and Goma.

**Additional file 3: Table 3.** Relationship between in-hospital mortality, sex, age, vital signs and Red Cross Wound Score (RCWS). Table of univariate and logistic regression analysis examining the relationship between in-hospital mortality, sex, age, vital signs RCWS grade and type for patients treated at ICRC’s hospitals in Peshawar and Goma.

## Data Availability

The datasets used and analysed during the current study are available from the corresponding author on reasonable request.

## Abbreviations

AIC: Akaike Information Criterion
AUC: Area under the receiver operating characteristic curve
CI: Confidence interval
ICRC: International Committee of the Red Cross
ISS: Injury Severity Score
KTS: Kampala Trauma Score
OR: Odds ratio
RCWS: Red Cross Wound Score
RTS: Revised Trauma Score
SD: Standard deviation
