# Supplementary material for: Predicting surgical resource consumption and in-hospital mortality in resource-scarce conflict settings: a retrospective study
Source: BMC Emerg Med. 2021 Aug 11;21:94. doi: 10.1186/s12873-021-00488-2 (PMC8359038; doi:10.1186/s12873-021-00488-2)
Supplement: Supplementary file 1 — Additional file 1: Table 1. Explanation on how to classify penetrating injuries according to Red Cross Wound Score (RCWS). [file 12873_2021_488_MOESM1_ESM.pdf]

Additional table 1 Explanation of Red Cross Wound Score (RCWS) [4]

RCWS is an anatomical scoring system that was designed by the International Committee of the Red Cross (ICRC) to quickly and relatively easily give guidance on a penetrating wound's significance. RCWS is based on size, tissue involved and location. Thus, RCWS is not dependent on the type of weapons or projectile that caused the wound. The following variables are included in the assessment of a wound according to RCWS:

E	Entry wound in centimetres
X	Exit wound in centimetres
C	Presence of cavity >2 fingers wide (0=No and 1=Yes)
F	Fracture (0=No fracture, 1=Simple fracture, hole or insignificant comminution and 2=Clinically significant comminution)
V	Vital structure (0=No penetration of vital structure, N=Penetration of the dura of the brain or spinal cord, A=Peritoneum, T=Pleura and H=Major peripheral vessels extending to the brachial or popliteal or carotid arteries)

Type	Grade 1	Grade 2	Grade 3
**ST**<br>Soft tissue wounds	Small simple wound<br><br>E+X<10cm<br>C0<br>F0<br>V0<br>**1ST**	Medium sized soft tissue wound<br>E+X<10cm<br>C1<br>F0<br>V0<br>**2ST**	Large soft tissue wound<br><br>E+X≥10cm<br>C1<br>F0<br>V0<br>**3ST**
**F**<br>Wounds with fractures	Small wound with simple fracture<br>E+X<10cm<br>C0<br>F1<br>V0<br>**1F**	Medium sized wound with important fracture<br>E+X<10cm<br>C0<br>F2<br>V0<br>**2F**	Large wound with significant fracture<br>E+X≥10cm<br>C1<br>F2<br>V0<br>**3F**
**V**<br>Vital wounds	Small wound threatening life<br><br>E+X<10cm<br>C0<br>F0<br>V=N OR T OR A OR H<br>**1V**	Medium sized wound threatening life<br>E+X<10cm<br>C1<br>F0<br>V= N OR T OR A OR H<br>**2V**	Large wound threatening life<br><br>E+X≥10cm<br>C1<br>F0<br>V= N OR T OR A OR H<br>**3V**
**VF**<br>Vital wounds with fractures	Small wound threatening limb or life<br>E+X<10cm<br>C0<br>F1<br>V= N OR T OR A OR H<br>**1VF**	Medium wound threatening limb or life<br>E+X<10cm<br>C1<br>F2<br>V= N OR T OR A OR H<br>**2VF**	Large wound threatening limb or life<br>E+X≥10cm<br>C1<br>F2<br>V= N OR T OR A OR H<br>**3VF**

4.Giannou C, Baldan M. War surgery: working with limited resources in armed conflict and other situations of violence. Vol 1. Geneva: International Committee of the Red Cross; 2019.
