# Supplementary material for: Predicting surgical resource consumption and in-hospital mortality in resource-scarce conflict settings: a retrospective study
Source: BMC Emerg Med. 2021 Aug 11;21:94. doi: 10.1186/s12873-021-00488-2 (PMC8359038; doi:10.1186/s12873-021-00488-2)
Supplement: Supplementary file 2 — Additional file 2: Table 2. Relationship between surgical resource consumption, sex, age, vital signs and Red Cross Wound Score (RCWS). Table of univariate and logistic regression analysis examining the relationship between surgical resource consumption, sex, age, vital signs, RCWS grade and type forpatients treated at ICRC’s hospitals in Peshawar and Goma. [file 12873_2021_488_MOESM2_ESM.pdf]

Additional table 2 Relationship between surgical resource consumption, sex, age, vital signs and Red Cross Wound Score (RCWS)

Surgical resource consumption	Univariate analysis			Logistic regression analysis			
	Complete cases Total=834			Complete cases Total=834		All cases[a] Total=1555[b]	
	Low Total=472 n (%)	High[c] Total=362 n (%)	P value	All confounders 1=High[c] Odds ratio (95% CI)	P value	All confounders 1=High[c] Odds ratio (95% CI)	P value
Sex			1.000				
Male	418	320 (88.4)		1		1	
Female	54 (11.4)	42 (11.6)		1.14 (0.68-1.90)	0.626	1.14 (0.79-1.63)	0.491
Age			0.710				
15-49 years	433	329 (90.9)		1		1	
>49	39 (8.3)	33 (9.1)		1.23 (0.70-2.17)	0.466	1.84 (1.26-2.70)	0.002
Time since injury			0.152				
0-6 hours	15 (3.2)	21 (5.8)		1		1	
7-24 hours	203	159 (43.9)		0.68 (0.30-1.54)	0.352	1.03 (0.52-2.06)	0.933
>24 hours	254	182 (50.3)		0.66 (0.29-1.52)	0.327	0.97 (0.49-1.95)	0.938
Systolic blood pressure			0.004				
>89 mmHg	464	341 (94.2)		1		1	
76-89	5 (1.1)	10 (2.8)		1.16 (0.33-4.11)	0.931	1.52 (0.61-3.75)	0.362
0-75	3 (0.6)	11 (3.0)		1.65 (0.44-6.20)	0.821	1.23 (0.51-2.97)	0.640
Respiratory rate			0.446				
10-29/min	458	347 (95.9)		1		1	
≤9	-	-		-	-	-	
≥30	14 (3.0)	15 (4.1)		0.88 (0.37-2.09)	0.772	0.72 (0.42-1.26)	0.222
Glasgow Coma Scale			0.077				
13-15	465	347 (95.8)		1		1	
9-12	4 (0.9)	10 (2.8)		1.09 (0.31-3.87)	0.896	1.01 (0.19-5.37)	0.811
6-8	2 (0.4)	4 (1.1)		1.60 (0.22-11.65)	0.644	0.85 (0.20-3.59)	0.825
≤5	1 (0.2)	1 (0.3)		0.84 (0.05-15.76)	0.906	0.90 (0.37-2.18)	0.985
RCWS grade			<0.001				
1 (simple)	266	83 (22.9)		1		1	
2 (medium)	185	178 (49.2)		2.60 (1.81-3.74)	<0.001	2.51 (1.93-3.26)	<0.001
3 (large)	21 (4.4)	101 (27.9)		11.60 (6.61-20.36)	<0.001	10.76 (7.41-15.63)	<0.001
RCWS type			<0.001				
Soft tissue	220	67 (18.5)		1		1	
Fracture	164	180 (49.7)		2.39 (1.62-3.54)	<0.001	2.12 (1.60-2.82)	<0.001
Threatening life	59 (12.5)	45 (12.4)		2.16 (1.27-3.67)	0.005	1.92 (1.31-2.83)	0.001
Threatening life/limb	29 (6.1)	70 (19.4)		4.67 (2.61-834)	<0.001	3.05 (2.04-4.54)	<0.001
Additional severe injuries[d]			<0.001				
No	439	288 (79.6)		1		1	
Yes	33 (7.0)	74 (20.4)		2.90 (1.83-4.89)	<0.001	2.43 (1.72-3.44)	<0.001

[a]Using imputed data for incomplete cases; [b]9 patients with unknown surgical resource consumption excluded; [c]Defined as ≥3 surgical procedures under anesthesia or ≥3 blood transfusions or limb amputation; [d]Corresponding to Abbreviated Injury Score ≥2
