# Supplementary material for: Predicting surgical resource consumption and in-hospital mortality in resource-scarce conflict settings: a retrospective study
Source: BMC Emerg Med. 2021 Aug 11;21:94. doi: 10.1186/s12873-021-00488-2 (PMC8359038; doi:10.1186/s12873-021-00488-2)
Supplement: Supplementary file 3 — Additional file 3: Table 3. Relationship between in-hospital mortality, sex, age, vital signs and Red Cross Wound Score (RCWS). Table of univariate and logistic regression analysis examining the relationship between in-hospital mortality, sex, age, vital signs RCWS grade and type for patients treated at ICRC’s hospitals in Peshawar and Goma. [file 12873_2021_488_MOESM3_ESM.pdf]

Additional table 3 Relationship between in-hospital mortality, sex, age, vital signs and Red Cross Wound Score (RCWS)

In-hospital mortality	Univariate analysis			Logistic regression analysis			
	Complete cases Total=834			Complete cases Total=834		All cases[a] Total=1559[b]	
	No Total=813 n (%)	Yes Total=21 n (%)	P value	All confounders 1=In-hospital mortality Odds ratio (95%CI)	P value	All confounders 1=In-hospital mortality Odds ratio (95% CI)	P value
Sex			1.000				
Male	719 (88.4)	19 (90.0)		1		1	
Female	94 (11.6)	2 (10.0)		0.82 (0.17-4.04)	0.815	1.38 (0.65-2.92)	0.395
Age			0.701				
15-49 years	743 (91.4)	19 (90.5)		1		1	
>49	70 (8.6)	2 (9.5)		1.48 (0.31-7.00)	0.620	1.31 (0.60-2.85)	0.494
Time since injury			0.010				
0-6 hours	33 (4.1)	3 (14.3)		1		1	
7-24 hours	354 (43.5)	8 (38.1)		0.32 (0.50-1.99)	0.222	0.37 (0.12-1.16)	0.089
>24 hours	426 (52.4)	10 (47.6)		0.41 (0.07-2.61)	0.347	0.65 (0.21-2.00)	0.451
Systolic blood pressure			0.033				
>89 mmHg	787 (96.8)	18 (85.7)		1		1	
76-89	13 (1.6)	2 (9.5)		2.46 (0.26-23.29)	0.433	4.63 (1.59-13.44)	0.005
0-75	13 (1.6)	1 (4.8)		1.04 (0.08-13.41)	0.978	2.34 (0.64-8.65)	0.194
Respiratory rate			0.163				
10-29/min	786 (96.7)	19 (90.5)		1		1	
≤9	-	-		-	-	1.90 (0.95-3.80)	0.070
≥30	27 (3.3)	2 (9.5)		3.02 (0.55-16.58)	0.203	3.00 (0.82-11.06)	0.097
Glasgow Coma Scale			<0.001				
13-15	796 (97.9)	16 (76.2)		1		1	
9-12	12 (1.5)	2 (9.5)		6.55 (1.00-43.13)	0.084	2.42 (0.27-21.47)	0.385
6-8	5 (0.6)	1 (4.8)		5.25 (0.44-62.71)	0.190	4.41 (0.78-24.75)	0.091
≤5	0	2 (9.5)		-	-	2.08 (0.62-6.98)	0.233
RCWS grade			0.656				
1 (simple)	338 (41.6)	11 (52.4)		1		1	
2 (medium)	355 (43.7)	8 (38.1)		0.56 (0.17-1.78)	0.323	1.37 (0.73-2.58)	0.323
3 (large)	120 (14.8)	2 (9.5)		0.37 (0.07-2.03)	0.250	1.39 (0.65-2.97)	0.400
RCWS type			0.007				
Soft tissue	283 (34.8)	4 (19.0)		1		1	
Fracture	339 (41.7)	5 (23.8)		1.48 (0.32-6.78)	0.612	1.12 (0.42-3.00)	0.820
Threatening life	98 (12.1)	6 (28.6)		3.88 (0.88-17.02)	0.073	10.72 (4.42-26.01)	<0.001
Threatening life/limb	93 (11.4)	6 (28.6)		2.89 (0.49-17.12)	0.241	8.43 (3.36-21.18)	<0.001
Additional severe injuries[c]			0.174				
No	711 (87.5)	16 (76.2)		1		1	
Yes	102 (12.5)	5 (23.8)		2.09 (0.67-6.52)	0.204	1.41 (0.75-2.65)	0.285

[a]Using imputed data for incomplete cases; [b]5 patients with unknown discharge status excluded; [c]Corresponding to Abbreviated Injury Score ≥2
